# Interspecific comparison of traffic noise effects on dove coo transmission in urban environments

**DOI:** 10.1038/srep32519

**Published:** 2016-08-31

**Authors:** Bao-Sen Shieh, Shih-Hsiung Liang, Yuh-Wen Chiu, Szu-Ying Lin

**Affiliations:** 1 Department of Biomedical Science and Environmental Biology, Kaohsiung Medical University, 100 Shihchuan 1st Road, Kaohsiung 807, Taiwan; 2 Department of Biotechnology, National Kaohsiung Normal University, 62 Sanchung Rd., Yanchao Township, Kaohsiung 824, Taiwan; 3 National Museum of Marine Biology and Aquarium, 2 Houwan Road, Checheng, Pingtung 944, Taiwan

## Abstract

Most previous studies concerning avian adaptation to anthropogenic noise have focused on songbirds, but few have focused on non-songbirds commonly found in urban environments such as doves. We conducted field playback-recording experiments on the perch-coos of five dove species, including four native Taiwan species (the spotted dove, *Spilopelia chinensis*, the oriental turtle-dove, *Streptopelia orientalis*, the red collared-dove, *Streptopelia tranquebarica*, and the emerald dove, *Chalcophaps indica*) and one species not native to Taiwan (the zebra dove, *Geopelia striata*) to evaluate the detection and recognition of dove coos in habitats with differing levels of traffic noise. Our results suggest that traffic noise has selected dominant urban species such as the spotted dove to temporally and spatially adjust cooing to reduce the masking effects of traffic noise and rare urban species such as the emerald dove to avoid areas of high traffic noise. Additionally, although the zebra dove had the highest coo frequency among the study species, its coos showed the highest detection value but not the highest recognition value. We conclude that traffic noise is an important factor in shaping the distribution of rare and dominant dove species in urban environments through its significant effects on coo transmission.

Fragkias *et al*.^1^ estimated that the urban human population of the world will increase to approximately 5 billion by 2030. Under this population pressure, urbanisation has expanded dramatically; urban ecosystems have become the primary habitats of humans and are the ecosystems most disturbed by human activities^2^. Anthropogenic noise, one of the most important by-products of human activity, has drawn attention in recent years (reviewed in ref. 3). Anthropogenic noise not only threatens animals indirectly at the species level but also at the community level (reviewed in ref. 4). Specifically, anthropogenic noise can limit the distribution and reproduction of sensitive species, disrupt interspecific relationships such as predator-prey interactions^5^, and cause changes to community structure^6^,^7^,^8^, resulting in a decrease in biodiversity^4^,^9^.

For birds, which rely on acoustic communication for functions such as predator alarm, mate attraction, and territorial defence^10^,^11^, reducing the masking effect of anthropogenic noise and transmitting acoustic signals through the noisy urban environments is vital for survival and reproduction^8^. Anthropogenic noise has been found to negatively affect the abundance of various bird species (e.g. ref. 12) and to shift community composition towards a few dominant species. Moreover, long-term or short-term signalling adaptations may occur that reduce the masking effects of background noise (reviewed in ref. 13). Among these signalling adaptations, several species of songbirds that learn their songs have been found to use frequency shifts in association with urban tolerance (reviewed in ref. 14). It has been suggested that high frequency songs are less affected by low-frequency anthropogenic noise^15^. Most of the previous studies on noise adaptation have focused on songbirds rather than non-songbirds.

In addition to the bias towards songbird species in previous noise adaptation studies, the heterogeneous structure of anthropogenic noise in urban habitats, which has characteristics similar to those of natural habitats^6^, has rarely been investigated. It is important to place greater emphasis on temporal and spatial heterogeneity in amplitude and other properties of noise in studies of anthropogenic noise effects on wildlife^16^. Traffic noise, the major sound source of anthropogenic noise in urban environments^9^, has been found to have various impacts on animals and, more broadly, on ecological communities^17^. Most of the energy in traffic noise tends to be concentrated below 3 kHz^6^,^18^; therefore, the masking effects of traffic noise are particularly pronounced for bird species that vocalise within that same frequency range^19^. Thus, for non-songbirds that vocalise in the frequency range below 3 kHz and have vocalisations with a rigidly programmed acoustic structure, signalling adaptations in response to traffic noise will strongly depend on the heterogeneous structure of noise such as amplitude, timing, and vertical stratification. Loud, continuous traffic noise will most likely lead to long-term adaptations^20^ or to animals avoiding the vicinity of the noise^19^. Instead of making short-term acoustic adjustments such as frequency shifts, non-songbird species residing in urban habitats are expected to show behavioural adjustments such as adjusting the timing of signalling or selecting habitats away from roads^8^ and, thus, reducing the noise amplitude.

In doves, perch-coos are considered functionally similar to the territorial songs of passerines^21^. Consequently, perch-coos have been suggested as having a function in long-range communication^22^, in which the intended receiver is a long distance away from the signaller and, usually, out of sight. In addition, perch-coos are basically innate and highly stereotyped^23^, species-specific^24^, and potentially effective signals for species isolation^21^. Doves rely heavily on perch-coos for reproduction. Nevertheless, doves coo at low frequencies^23^ that are within the primary frequency range of traffic noise. Thus, the transmission of perch-coos that reduce the masking effect of traffic noise is critical for doves in urban environments. To date, there have been many studies on noise adaptation by songbirds but few involving non-songbirds such as doves, even though many dove species inhabit urban environments globally^25^.

In the present study, we address the question of how the masking effect of traffic noise might affect signal transmission in dove species, including both dominant urban species and rare urban species that are rarely observed in the vicinity of traffic noise as well as introduced urban species that are in the process of becoming established in urban environments. The dove species in this study included four native Taiwan species (the spotted dove, the oriental turtledove, the red collared-dove, and the emerald dove) and one species introduced to Taiwan, the zebra dove. First, we quantified the amplitude and acoustic properties of traffic noise under different conditions with regard to temporal heterogeneity (timing of traffic) and spatial heterogeneity (habitats). Second, because the most important question in signal transmission is whether a signal can be detected and recognised by the intended receiver^26^, we aimed to evaluate the detection and recognition of perch-coos of dove species under different noise conditions (timing of traffic and habitats) using playback-recording experiments.

## Results

### Comparative analysis of test coo sounds in five dove species

The acoustic characteristics (Supplementary Table S1) of 20 test coo sounds for each study species are summarised in Supplementary Table S2, and spectrograms for a representative test coo sound for each species are shown in Supplementary Fig. S1 (Supplementary Audio 1,2,3,4,5). All measurements showed significant differences among species (Kruskal-Wallis Test, p < 0.05; Supplementary Table S2). The zebra dove had the highest frequency-related measurements among the five species and the spotted dove had the second highest values, including peak frequency (PF), minimum frequency (MinF), maximum frequency (MaxF), and frequency distribution (Q1, Q2, and Q3). These two species did not differ significantly in bandwidth or entropy measurements. Spotted doves, oriental turtle-doves, and red collared-doves had similar ratios of harmonic to nonharmonic energy (HNR) in their coo sounds. The emerald dove, which had the most tonal structures in its coos, had the lowest values for entropy, bandwidth, and pureness (Supplementary Table S2).

### Maximum sound levels and sound characteristics of six noise conditions

**Figure 1:**
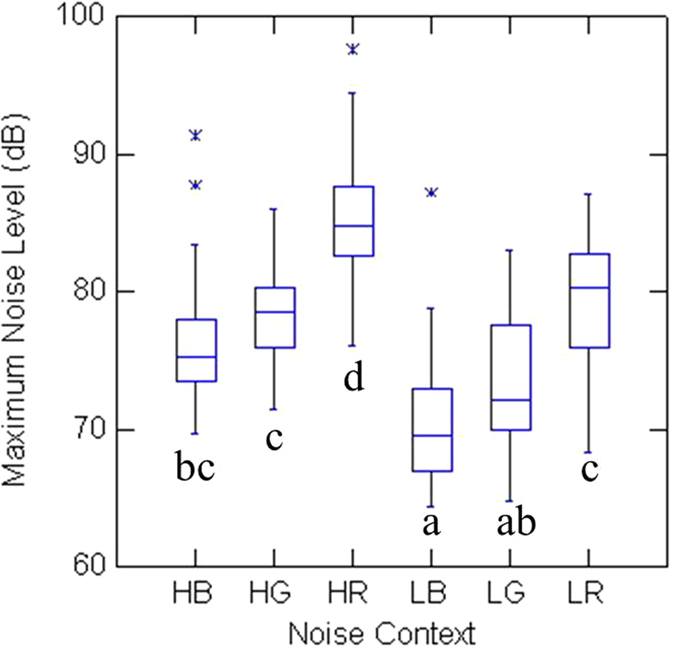
Box-plots of maximum sound levels (dB) under different noise conditions: low traffic building top (LB), low traffic green land (LG), low traffic roadside (LR), high traffic building top (HB), high traffic green land (HG), and high traffic roadside (HR). Different letters indicate significant differences (Wilcoxon signed ranks test, p < 0.01) between the two conditions. Asterisks represent extreme values.

There were significant differences in maximum sound levels among the six noise conditions (Friedman test statistic = 59.9, p < 0.01, df = 5). The maximum noise levels were highest in high traffic roadside conditions (HR); and lowest in low traffic building tops (LB) (Fig. 1). However, there were non-significant differences between low traffic building tops (LB) and low traffic green lands (LG) (Wilcoxon signed rank test, p = 0.08), between high traffic building tops (HB) and high traffic green lands (HG) (Wilcoxon signed rank test, p = 0.38), and between low traffic roadsides (LR) and high traffic green lands (HG) (Wilcoxon signed rank test, p = 0.47).

In an examination of the power spectra of background noise under the six noise conditions (Supplementary Fig. S2), the masking effects of background noise on the frequency ranges of the five study species (0.3 kHz–1.4 kHz) were greatest in high traffic roadside conditions (HR) and smallest in low traffic building tops (LB). Furthermore, in comparing the acoustic characteristics of background sounds recorded from the six noise conditions, all measurements except minimum frequency (MinF) and the ratio of harmonic to nonharmonic energy (HNR) showed significant differences among noise conditions (Supplementary Table S3). In particular, the background noise on high traffic roadsides had the highest maximum frequency (MaxF), highest energy distribution (Q1, Q2, and Q3), highest entropy, highest bandwidth and highest pureness. Although the four frequency characteristics—peak frequency (PF), maximum frequency (MaxF), Q1, and bandwidth—were lowest in low traffic building tops (LB), non-significant differences were found between building tops (B) and green lands (G) whether in high traffic (H) or low traffic conditions (L).

### PCA of test coo sounds, received coo sounds and background noises

Two principal components were derived from the correlation matrix of normalised acoustic variables by Principal Component Analysis (PCA) (Table 1). The first two components (PC1 and PC2) accounted for 85.1% of the total variation. PC1 accounted for 52.9% of the total variation, with a higher value of PC1 corresponding to a higher frequency distribution (Q1, Q2, and Q3) and a higher entropy. PC2 accounted for 32.2% of the total variation, with a higher value of PC2 corresponding to a higher pureness value and a lower peak frequency (PF) (Table 1).

**Figure 2:**
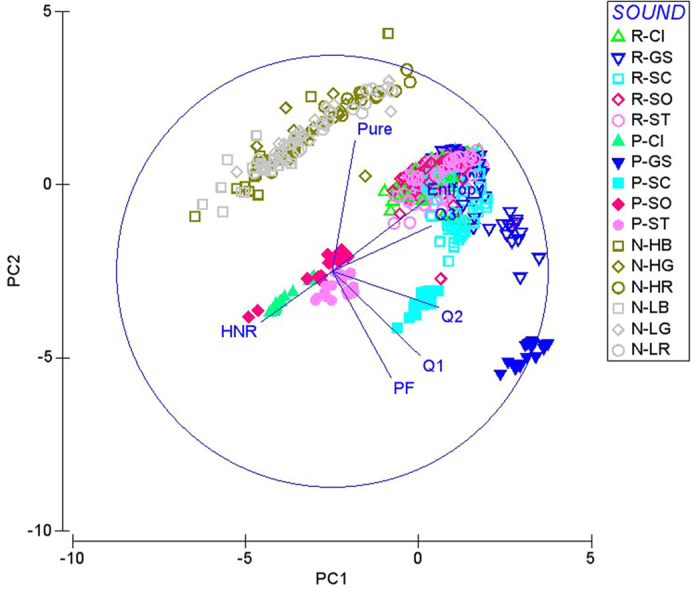
A bi-dimensional diagram of the principal component analysis using the seven acoustic variables from the spectrograms of background noises (N), and test sounds (P), received sounds (R) of the five dove species: the emerald dove (CI), the zebra dove (GS), the spotted dove (SC), the oriental turtle-dove (SO), and the red collared-dove (ST).

When plotting PC1 against PC2, the background noises were separated from the test coo sounds and received coo sounds, whereas the test coo sounds overlapped with the received coo sounds (Fig. 2).

**Figure 3:**
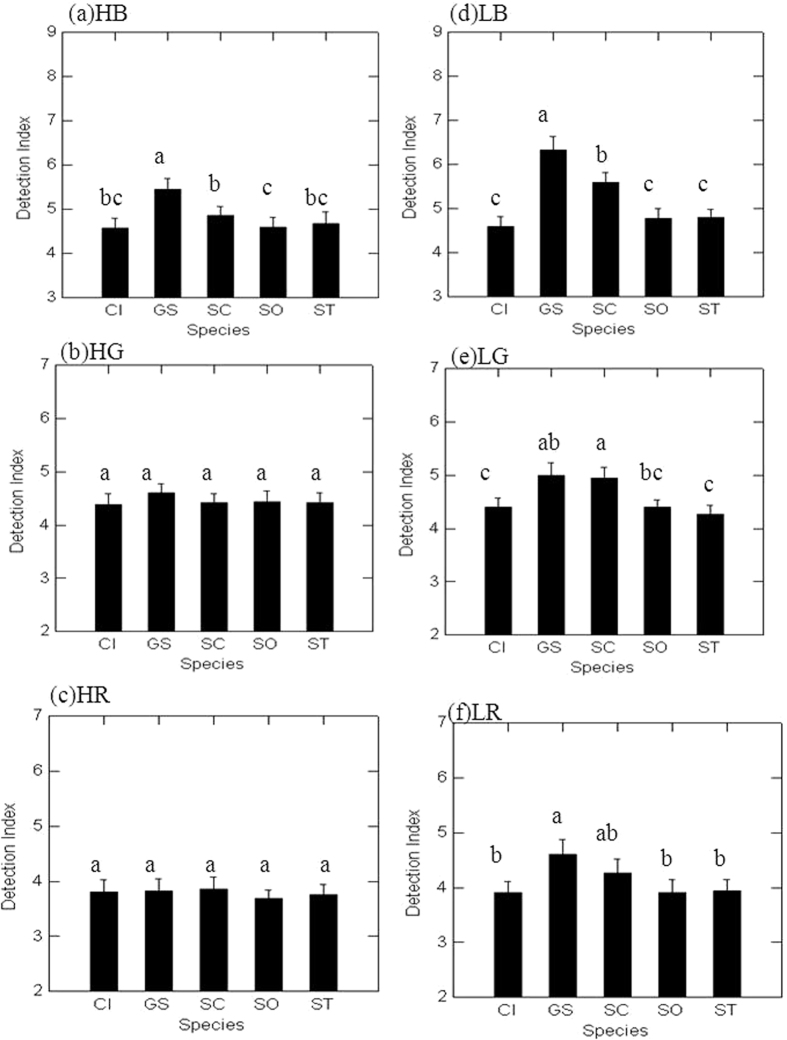
The comparison of detection indices (mean ± SE) among the five dove species, the emerald dove (CI), the zebra dove (GS), the spotted dove (SC), the oriental turtle-dove (SO), and the red collared-dove (ST), under different noise conditions: (**a**) high traffic building top (HB), (**b**) high traffic green land (HG), (**c**) high traffic roadside (HR), (**d**) low traffic building top (LB), (**e**) low traffic green land (LG), and (**f**) low traffic roadside (LR). Different letters indicate significant differences (paired t-test, Bonferroni-adjusted p-value < 0.05) between the two species.

In the LB (low traffic building tops) noise conditions, which had the lowest noise levels, coo sounds of zebra doves had the greatest detection index values, and those of spotted doves had the second greatest values, whereas in the HR (high traffic roadside) noise conditions, which had the highest noise levels, non-significant differences were found among the five dove species (Fig. 3).

**Figure 4:**
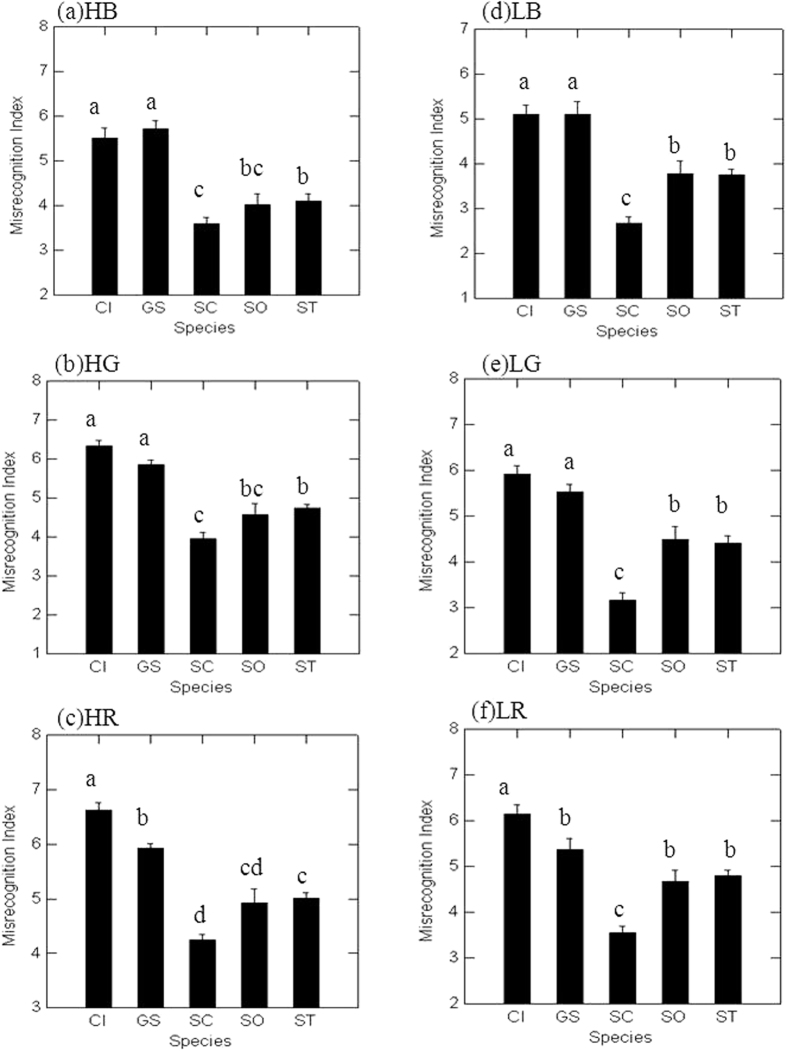
The comparison of misrecognition indices (mean ± SE) among the five dove species, the emerald dove (CI), the zebra dove (GS), the spotted dove (SC), the oriental turtle-dove (SO), and the red collared-dove (ST), under different noise conditions: (**a**) high traffic building top (HB), (**b**) high traffic green land (HG), (**c**) high traffic roadside (HR), (**d**) low traffic building top (LB), (**e**) low traffic green land (LG), and (**f**) low traffic roadside (LR). Different letters indicate significant differences (paired t-test, Bonferroni-adjusted p-value < 0.05) between the two species.

Under all noise conditions, in particular, in low traffic conditions—whether building tops (LB), green lands (LG) or roadsides (LR)—the coos of spotted doves had significantly lower misrecognition index values than those of the other four species (Fig. 4). Furthermore, in contrast to the detection index, the coos of the zebra dove, the species with the highest detection index, did not show the lowest misrecognition index values; instead, the zebra dove and the species with the most tonal coos, the emerald dove, had coos with the highest misrecognition index values on building tops (HB and LB) and green lands (HG and LG) (Fig. 4).

## Discussion

Our analyses of background noise revealed that amplitude and other acoustic properties differed significantly among noise conditions. On high traffic roadsides, the primary source of noise was traffic; traffic noise had the highest amplitude and was characterised by a maximum frequency of 1202.7 Hz and a bandwidth of 1192.5 Hz, which were the highest values among all conditions. Furthermore, based on our analyses of the power spectra of background noise, we determined the masking effects of noise by examining the amount of frequency overlap between signals (coos) and background noise^27^ (i.e., the energy coverage between 0.3–1.4 kHz) (Supplementary Fig. S2). We found that the masking effects of noise varied with noise conditions and were greatest on high traffic roadsides. In comparison with other studies (e.g. ref. 28), the maximum frequency of our traffic noise (1202.7 Hz) was lower than that (2,556 Hz) found in high traffic sites (near interstate highway I-80 in North America). This might be because we chose to investigate the frequency range below 2 kHz by analysing at a sampling rate of 4 kHz. The 2 kHz frequency range was chosen based on the coo frequency range of our study species and the sound frequency range of anthropogenic noise^29^.

Our results also revealed that building tops, which have rarely been investigated in previous studies, had noise levels as low as green lands both under high traffic and low traffic conditions. Furthermore, the masking effects of background noise were smallest on low traffic building tops. In other words, avoiding the impact of traffic noise by leaving the vicinity of traffic noise vertically (from roadsides to building tops) is as effective as leaving the vicinity horizontally (from roadsides to green lands)—and sometimes even better.

Our study found that the spotted dove, the commonest species in urban environments, had the greatest detection index and the lowest misrecognition index compared with the other three native species, which were rarely observed or heard at our study sites. This finding would be expected if the masking effect of traffic noise has played a significant role in the signal transmissions of doves in urban environments. Furthermore, with regard to the low frequency and stereotypic nature of perch-coos, we expected that the main urban resident, the spotted dove, would adjust its cooing temporally and spatially to reduce the masking effects of traffic noise based on our analyses of noise amplitudes. In other words, it is best to coo at times when traffic is infrequent and on building tops, which had the lowest amplitudes of traffic noise, rather than during high traffic periods and near roadsides, which had the highest amplitudes of traffic noise. This expectation is concordant with the observation that the yellow-billed cuckoo (*Coccyzus americanus*) and the white-breasted nuthatch (*Sitta carolinensis*) that vocalise at a peak frequency of less than 2 kHz demonstrate significant spatial shifts to avoid the effects of high traffic noise^19^.

Although the zebra dove, the introduced species, had the highest coo frequency among the study species, its coos showed the highest detection value but not the highest recognition value. This finding indicates that the high frequency coos of zebra doves could be detected but that their “acoustic patterns” were easily lost after being masked by traffic noise. This finding is not consistent with the noise-dependent song frequency adaptation suggested by previous studies^13^,^14^. Although traffic noise can affect both the detection and recognition of a signal by intended receivers^30^, we suggest that its effects on detection and recognition are not both frequency-dependent, in particular when the signal frequency is within the general range of traffic noise (i.e., less than 2 kHz).

Our results are also contradictory to a study of the great tit (*Parus major*), in which signals with sound energy concentrated within a narrow frequency range were generally easier to detect than signals spread over a wide frequency range^31^. The rare urban species, the emerald dove, had the highest pureness values and the narrowest frequency range of coos; both the detection and recognition of its coos were the worst among all the study species. This discrepancy might be because the emerald dove had the lowest coo frequency and therefore suffered the greatest masking effects from low frequency noise.

In our study, we did not measure the field response of conspecifics to the playback coos and, therefore, could not validate the perceptual relevance of acoustic features of the received sounds^32^. In other words, a high acoustic similarity between the playback coos and the received sounds, defined as high recognition (low misrecognition index) in our study, does not guarantee a high response from conspecifics. However, a low acoustic similarity between the playback coos and the received sounds, defined as low recognition (high misrecognition index) in our study, is expected to be less likely to obtain a response from conspecifics^32^,^33^. The coos of the rare urban species, the emerald dove, had the lowest/worst recognition in all habitats among all the study species. Cooing without the receivers’ response because of poor recognition could impair the reproductive functions of coos and decrease fitness for emerald doves in urban environments. Therefore, we suggest that poor recognition due to noise effects is an important factor that contributes to the rarity of emerald doves in urban environments.

The results of our playback-recording experiments matched the predictions of the hypothesis: the masking effect of traffic noise plays a significant role in the signal transmission of dove species in urban environments. Traffic noise has selected for dove species such as spotted doves, which have perch-coo adaptations, and negatively selected for dove species such as emerald doves, which leave the vicinity of the noise. Rather than making short-term acoustic adjustments such as frequency shifts, non-songbird urban residents such as spotted doves were expected to exhibit behavioural adjustments such as adjusting the timing of signalling (i.e., cooing at low traffic times and/or selecting vertical habitats such as building tops away from roads) and, thus, reducing the masking effects of traffic noise.

## Methods

### Study area

We conducted playback-recording experiments at 20 different sites (N22.60~N22.67, E120.28~E120.33) in the urban area of Kaohsiung City in the south of Taiwan in June 2015.

The spotted dove is the commonest dove species in the urban area of Kaohsiung City, followed by the red collared-dove and the oriental turtle-dove, whereas the emerald dove is rarely observed and occurs only within the Shoushan Nature Park in Kaohsiung City. The zebra dove is an introduced species in Taiwan; it was imported into Taiwan through the pet trade from Southeast Asia. The zebra dove was first sighted in the field in Kaohsiung City in 2006; since then, it has established populations in only two city parks in Kaohsiung City. During our experimental periods, we heard the natural coos of spotted doves at all study sites and observed the presence of the red collared-dove at one of the study sites. The zebra dove, the oriental turtle-dove, and the emerald dove were neither observed nor heard at any of the 20 study sites.

### Field recordings and editing to generate test coo sounds

All perch-coos for the test sounds used in the experiments were recorded from free-living doves in Taiwan, including Taipei City, Kenting National Park, Kaohsiung City, and Green Island. Perch-coos, which are produced from a high perch without conspecifics nearby and, usually, out of sight of a potential receiver^34^, were identified and recorded using a Denon Portable IC Recorder (DN-F20R) equipped with a Sennheiser ME67 unidirectional microphone. We selected high-quality coos with clear acoustic structures on spectrograms as the source sounds for each dove species. The source sounds came from nine cooing bouts of four individuals for emerald doves, 21 cooing bouts of seven individuals for zebra doves, 57 cooing bouts of 25 individuals for spotted doves, 22 cooing bouts of seven individuals for oriental turtle-doves, and 18 cooing bouts of 12 individuals for red collared-doves. These source sound recordings were digitised and filtered using Avisoft-SASLab Pro software v5.2.05. Because the coos of the studied dove species were observed to have different frequency ranges, different band-pass filters were used to remove noise components from the recordings for the different species. The band-pass filters for each species were set as follows: 0.7–1.4 kHz for zebra doves, 0.4–1.1 kHz for spotted doves, 0.3–0.7 kHz for oriental turtle-doves, 0.3–0.7 kHz for red collared-doves, and 0.3–0.55 kHz for emerald doves. Twenty 20-second test sounds for each dove species were made by editing the source sounds according to the following criteria. All coos from the source sounds were used in the test sounds when possible, and coos from the same bouts were edited into in the same test sounds. A coo was defined as the smallest stereotypic repetition of a similar element sequence^23^. The number of coos was adjusted to maintain the total duration of coos in a test sound at approximately 10 seconds with silent intervals comprising the remaining 10 seconds.

The edited test sounds for each dove species were first standardised for amplitude using Audacity software v.2.0.6 (effect menu: normalise) and then merged into 20 test sound files randomly corresponding to 20 sites. Each test sound file consisted of five test sounds, one from each dove species, arranged in a Latin Square design. After merging the edited test sounds, we standardised the amplitude of the test sound files a second time. In the subsequent playback-recording experiments, the test coo sound files were broadcast and recorded at each study site under a 5 × 2 × 3 factorial design, i.e., with the five study species, the two traffic conditions (L, low traffic and H, high traffic), and the three habitat types (B, building tops, at least 20 m high above the ground; G, green lands, at least 20 m away from the roadways; and R, roadsides, within 5 m of roadways).

### Playback-recording experiment

We used a Marantz Recorder (PMD 661 MKII) connected to a wireless speaker (MIPRO MA-101, frequency response 50–15000 Hz ± 3 dB) for the playback. The speaker was mounted 1.5 m above the ground on a tripod. Playback-recording experiments were conducted from 05:00 to 06:45 h, a period corresponding to low traffic conditions, and from 07:30 to 09:15 h, a period corresponding to high traffic conditions. Before each trial began, the maximum noise level (in decibels) was measured for one minute at the position of the microphone using a sound level metre (TES-1350; slow response, C-weighting function) held horizontally at a height of approximately 1.5 m and rotated 360° clockwise. At the beginning of each trial, a 20-second recording was made of the background noise. A test sound file of the coos of five dove species was then played back at a standardised volume with a sound-pressure level of approximately 90 dB at the speaker and recorded with a Denon Portable IC Recorder (DN-F20R) connected to a shotgun microphone (Sennheiser ME67) that was placed on a stand 1.5 m above the ground and oriented towards the broadcasting speaker at a distance of 10 m. Each test sound file had six trials with recordings at a corresponding study site: two traffic conditions (H, L) x three habitat types (B, G, R), i.e., each test sound file had one trial (one recording) in each of the six noise conditions (HB, LB, HG, LG, HR, and LR). These recordings supplied the received sounds for the data analysis.

### Data analysis

We changed the format of the recordings from a sampling frequency of 48 kHz to 4 kHz and produced spectrograms with the following settings: FFT = 512, frame size = 100%, hamming window, overlap = 87.5%, frequency resolution = 8 Hz, and time resolution = 16 ms. We quantified 10 acoustic variables (Supplementary Table S1) on the spectrograms using the Automatic Parameter Measurements function in Avisoft-SASLab Pro software v5.2.05.

For the recordings of received sounds but not the background noise, we removed the noise components using a band-filter of 0.3–1.4 kHz, which was the observed frequency range of coos for our five study species and was assumed to be their perceptually tuned frequency range^35^. However, with these cut-off frequencies, the minimum and maximum frequencies of the sounds were unavoidably close to 0.3 kHz and 1.4 kHz, respectively. Therefore, the minimum frequency, maximum frequency and bandwidth of sounds were not used for the subsequent principal component analysis (PCA). Acoustic comparisons of background noise, test sounds, and received sounds were then explored using the PCA of those seven measured variables after a normalised transformation of each variable to a mean of zero and unit standard deviation (software PRIMER 6, version 6.1.5). We retained all components with eigenvalues greater than one.

Two indices, the detection index and the misrecognition index, were derived from PC1 and PC2. We defined the detection index as a measure of the separation between a signal and background noise and the misrecognition index as a measure of the ability to identify a signal from received signals. Therefore, on the bi-dimensional plotting of PC1 against PC2, the detection index was calculated by measuring the distance between the background sound sample and the corresponding received coo sample recorded under the same noise condition at the same site. A higher detection index indicates that the received coo sound is substantially different from the background noise; thus, it is more likely to be detected. The misrecognition index was calculated by measuring the distance between the test coo sample and the corresponding received coo sample recorded under the same noise condition at the same site. A higher misrecognition index indicates that the received coo is substantially different from the test coo; thus, it is less likely to be recognised.

For untransformed variables of acoustic measurements, we assessed group differences by performing a Kruskal-Wallis test (two-tailed, with a significance level of 0.05) and then made pairwise comparisons using a Mann-Whitney U test (two-tailed, with a significance level of 0.01 to adjust for multiple comparisons). For maximum noise levels, we compared the six noise conditions by performing a two-way Friedman ANOVA with the sites as groups (two-tailed, with a significance level of 0.05) and then made pairwise comparisons using a Wilcoxon signed rank test (two-tailed, with a significance level of 0.01 to adjust for multiple comparisons). For the two indices under each noise condition, we assessed interspecific differences under each noise condition by performing two-way ANOVA with sites as groups (two-tailed, with a significance level of 0.05) and then made pairwise comparisons using a paired t-test (two-tailed, with a significance level of 0.05 for the Bonferroni-adjusted p-value). The PCAs were performed using PRIMER 6.0, and nonparametric tests were performed using SYSTAT 11.

## Additional Information

**How to cite this article**: Shieh, B.-S. *et al*. Interspecific comparison of traffic noise effects on dove coo transmission in urban environments. *Sci. Rep.* **6**, 32519; doi: 10.1038/srep32519 (2016).

## Supplementary Material

Supplementary Information

Supplementary Audio 1

Supplementary Audio 2

Supplementary Audio 3

Supplementary Audio 4

Supplementary Audio 5

## Figures and Tables

**Table 1:** Eigenvalues, percentage of variance explained, and coefficients (eigenvectors) of the normalised variables in the first two principal components extracted from the seven acoustic variables.

	Principal component	
	PC1	PC2
Eigenvalue	3.7	2.3
% Variance	52.9	32.2
**Variable**	**Coefficient (eigenvector)**	
PF	0.274	−0.496
Q1	0.408	−0.392
Q2	0.493	−0.167
Q3	0.460	0.211
Entropy	0.427	0.326
HNR	−0.331	−0.236
Pureness	0.105	0.605

Absolute values of coefficients greater than 0.4 are highlighted. Descriptions of the variables are provided in Supplementary Table S1.
