# Supplementary material for: Interspecific comparison of traffic noise effects on dove coo transmission in urban environments
Source: Sci Rep. 2016 Aug 31;6:32519. doi: 10.1038/srep32519 (PMC5006167; doi:10.1038/srep32519)
Supplement: Supplementary Information [file srep32519-s1.pdf]

Interspecific comparison of traffic noise effects on dove coo transmission in urban environments

Bao-Sen Shieh[1*], Shih-Hsiung Liang[2], Yuh-Wen Chiu[3], and Szu-Ying Lin[1]

Table S1. Descriptions of the acoustic variables measured on spectrograms.

Abbreviation	Description
PF	Peak frequency: the frequency of the maximum amplitude
MinF	The minimum frequency
MaxF	The maximum frequency
Q1	Below this frequency is 25% of the total energy
Q2	Below this frequency is 50% of the total energy
Q3	Below this frequency is 75% of the total energy
Entropy	The ratio of the geometric mean to the arithmetic mean of the spectrum.
HNR	The ratio of harmonic to nonharmonic energy
Bandwidth	Difference between the maximum frequency and the minimum frequency
Pureness	Difference between Q3 and Q1

Table S2. Mean (± SE) acoustic measurements (n = 20 for each species) on spectrograms of test sounds of five dove species. Asterisks indicate statistically significant differences among species (H: Kruskal-Wallis test statistic, df = 4; **: p < 0.01). Different superscript letters indicate significant differences (Mann-Whitney U test, df = 1, p < 0.01) between the two species.

	Species					
Variable	*Geopelia striata*	*Spilopelia chinensis*	*Streptopelia tranquebarica*	*Streptopelia orientalis*	*Chalcophaps indica*	H
PF	[a]1163.8 ± 5.6	[b] 772.9 ± 8.1	[c] 514.1 ± 6.6	[d] 428.5 ± 9.6	[d] 415.8 ± 1.7	88.8**
MinF	[a] 831.3 ± 9.9	[b] 471.7 ± 8.6	[c] 361.6 ± 9.0	[d] 300.0 ± 1.6	[c] 390.0 ± 2.8	89.6**
MaxF	[a] 1367.9 ± 8.3	[b] 1008.0 ± 8.8	[c] 681.5 ± 8.6	[c] 680.1 ± 8.1	[d] 450.7 ± 4.6	90.7**
Q1	[a] 1019.7 ± 4.3	[b] 683.6 ± 4.0	[c] 485.4 ± 6.7	[e] 404.0 ± 1.5	[d] 414.1 ± 2.2	92.6**
Q2	[a] 1128.4 ± 5.5	[b] 765.6 ± 2.4	[c] 525.8 ± 6.4	[d] 466.1 ± 1.8	[e] 424.1 ± 2.4	95.2**
Q3	[a] 1203.3 ± 5.3	[b] 838.2 ± 2.4	[c] 569.1 ± 6.8	[d] 525.8 ± 2.9	[e] 452.5 ± 11.5	88.4**
Entropy	[a] 0.620 ± 0.003	[a] 0.605 ± 0.006	[c] 0.462 ± 0.009	[b] 0.516 ± 0.006	[d] 0.240 ± 0.013	89.3**
HNR	[ab] 13.3 ± 1.4	[bc] 11.6 ± 0.9	[bc] 11.1 ± 0.8	[c] 11.0 ± 2.7	[a] 14.8 ± 0.5	19.8**
Bandwidth	[a] 536.7 ± 13.0	[a] 536.3 ± 12.3	[c] 320.0 ± 10.4	[b] 380.1 ± 9.3	[d] 60.8 ± 5.6	87.2**
Pureness	[a] 183.6 ± 2.1	[b] 154.6 ± 4.0	[d] 83.8 ± 3.2	[c] 121.8 ± 2.5	[e] 38.3 ± 9.8	84.8**

Table S3. Mean (± SE) acoustic measurements on spectrograms of background noise under different conditions (n = 20 for each condition). Asterisks indicate statistically significant differences among conditions (H: Kruskal-Wallis test statistic, df = 5, *p < 0.05, **p < 0.01). Different superscript letters indicate significant differences (Mann-Whitney U test, df = 1, *p* < 0.01) between the two conditions.

	Low Traffic			High Traffic			
	Building top	Greenland	Roadside	Building top	Greenland	Roadside	H
PF	[a]45.5 ± 7.5	[ab]59.6 ± 4.9	[b]78.5 ± 7.4	[a]51.8 ± 5.8	[ab]102.9 ± 46.3	[ab]73.6 ± 7.4	14.8*
MinF	9.4 ± 0.8	11.4 ± 0.9	11 ± 1.4	14.2 ± 2.7	10.2 ± 1.1	10.2 ± 1.3	3.8
MaxF	[a]480.3 ± 50.6	[a]728.4 ± 107.3	[b]1095.6 ± 113.1	[a]593.0 ± 92.4	[ab]735.4 ± 88.2	[c]1202.7 ± 117.6	34.3**
Q1	[a]82.7 ± 5.6	[a]111.6 ± 7.2	[b]134.7 ± 8.4	[a]91.0 ± 3.1	[ab]104.9 ± 5.5	[c]146.8 ± 8.2	48.8**
Q2	[a]199.5 ± 13.1	[bc]272.9 ± 23.8	[c]333.2 ± 23.9	[ab]231.6 ± 25.4	[bc]256.6 ± 15.0	[d]383.8 ± 28.5	39.0**
Q3	[a]557.2 ± 32.4	[b]745.2 ± 31.7	[b]838.7 ± 43.3	[a]623.4 ± 60.9	[b]748.9 ± 31.3	[c] 939.7 ± 35.5	47.5**
Entropy	[a]0.504 ± 0.018	[b]0.59 ± 0.018	[b]0.653 ± 0.221	[ac]0.517 ± 0.017	[c]0.580 ± 0.015	[d]0.693 ± 0.024	44.5**
HNR	14.4 ± 1.2	11.6 ± 1.1	10.4 ± 0.8	12.9 ± 1.6	11.3 ± 1.3	11.2 ± 0.97	8.5
Bandwidth	[a]470.9 ± 50.3	[a]717.0 ± 107.4	[b]1084.6 ± 113.5	[a]578.8 ± 93.5	[ab]725.2 ± 88.4	[c]1192.5 ± 118.2	34.2**
Pureness	[a]474.6 ± 31.5	[b]633.6 ± 27.6	[b]704 ± 35.6	[a]532.4 ± 60.5	[b]643.2 ± 29.9	[c]792.9 ±792.91.2	45.4**

Fig. S1 Spectrograms of representative test coo sounds for five dove species, *Chalcophaps indica* (Supplementary audio 1), *Geopelia striata* (Supplementary audio 2), *Spilopelia chinensis* (Supplementary audio 3), *Streptopelia orientalis* (Supplementary audio 4), and *Streptopelia tranquebarica* (Supplementary audio 5). The settings were sampling frequency = 4 kHz, FFT = 512, hamming window, overlap = 87.5%.

Chalcophaps indica

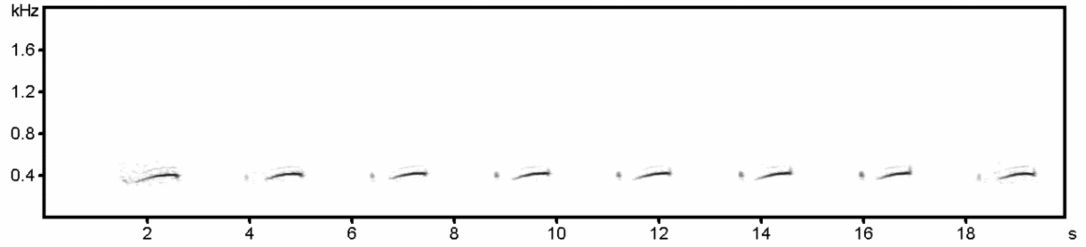

Geopelia striata

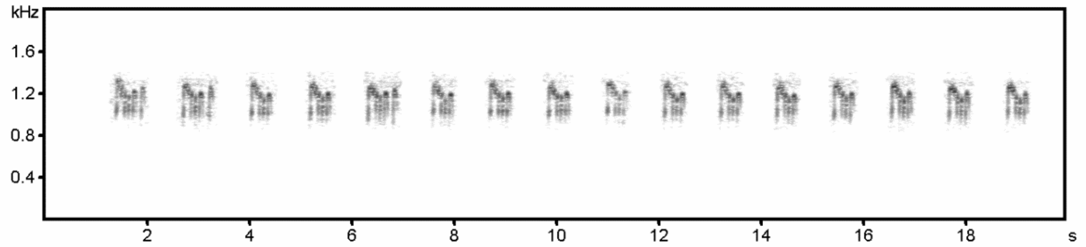

Spilopelia chinensis

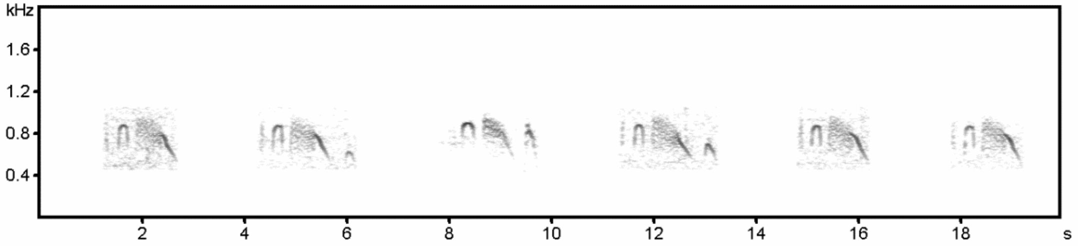

Streptopelia orientalis

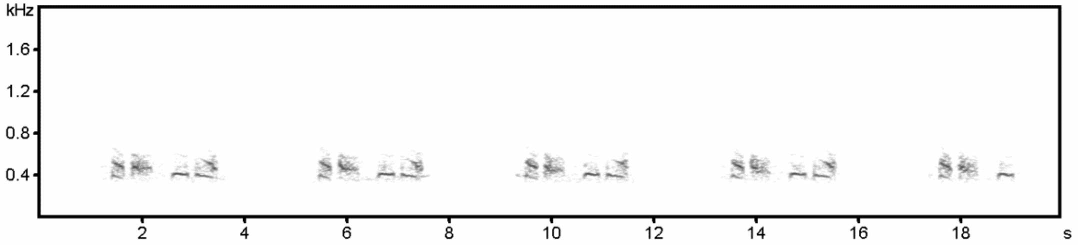

Streptopelia tranquebarica

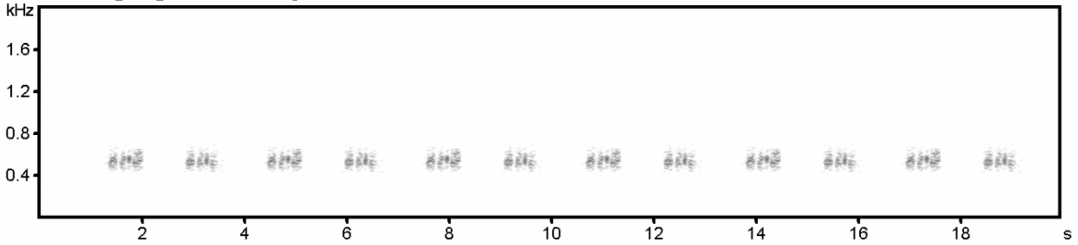

Fig. S2 Averaged power spectra of background noise under different noise conditions: (a) high traffic building top (HB), (b) high traffic green land (HG), (c) high traffic roadside (HR), (d) low traffic building top (LB), (e) low traffic green land (LG), and (f) low traffic roadside (LR). A 20-second sound was sampled at each site under each condition. The averaged power spectrum for each noise condition was made by compiling all sampled sounds from the 20 sites. The settings were sampling frequency = 4 kHz, FFT = 512, hamming window, overlap = 87.5%. Two vertical lines indicate the upper bound (1.4 kHz) and lower bound (0.3 kHz) of the coo frequency range of the study doves; the larger the area between the two vertical lines on the power spectrum, the greater the masking effect of the background noise.

(a) high traffic building top (HB)

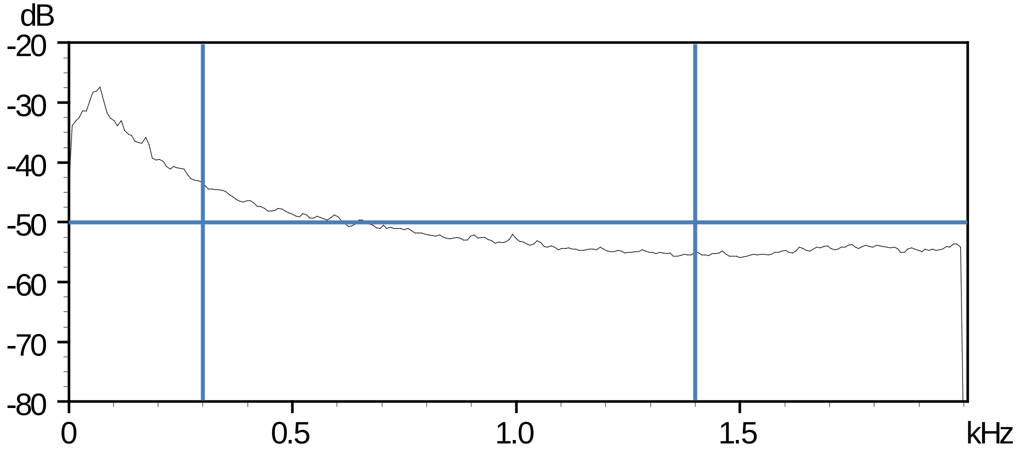

(b) high traffic green land (HG)

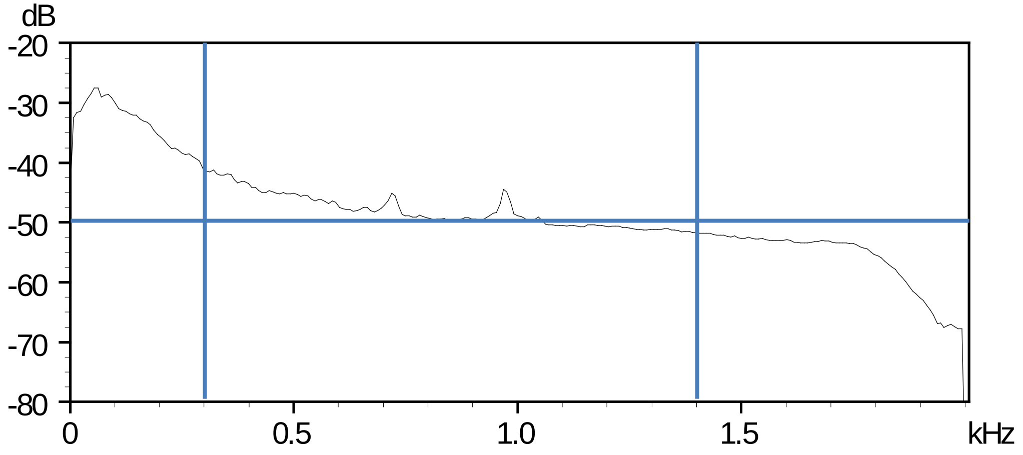

(c) high traffic roadside (HR)

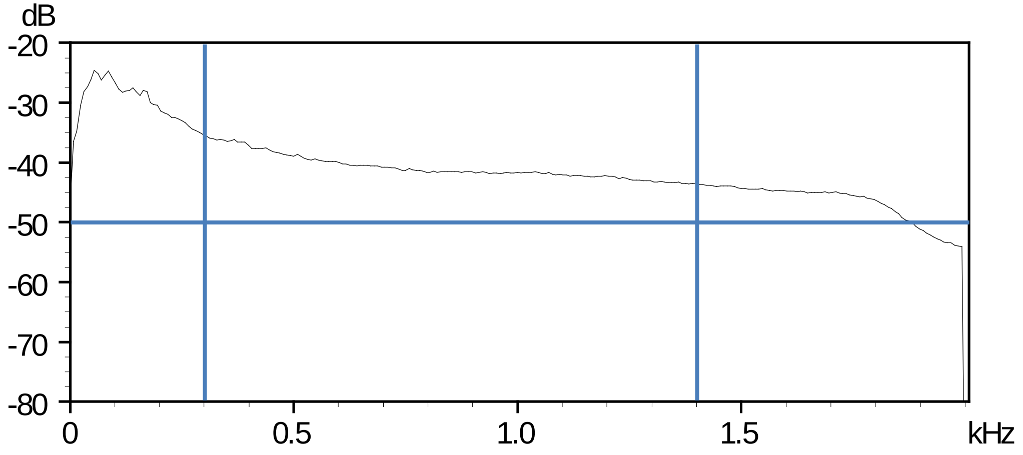

(d) low traffic building top (LB)

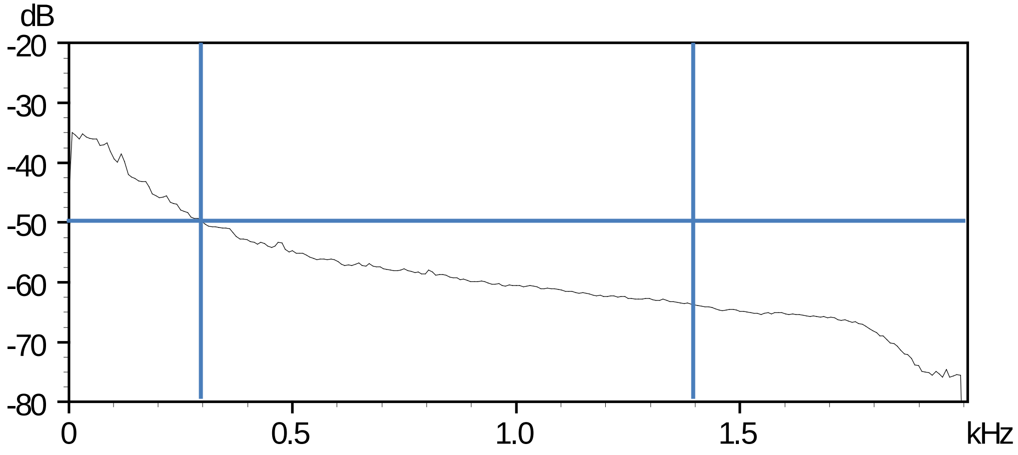

(e) low traffic green land (LG)

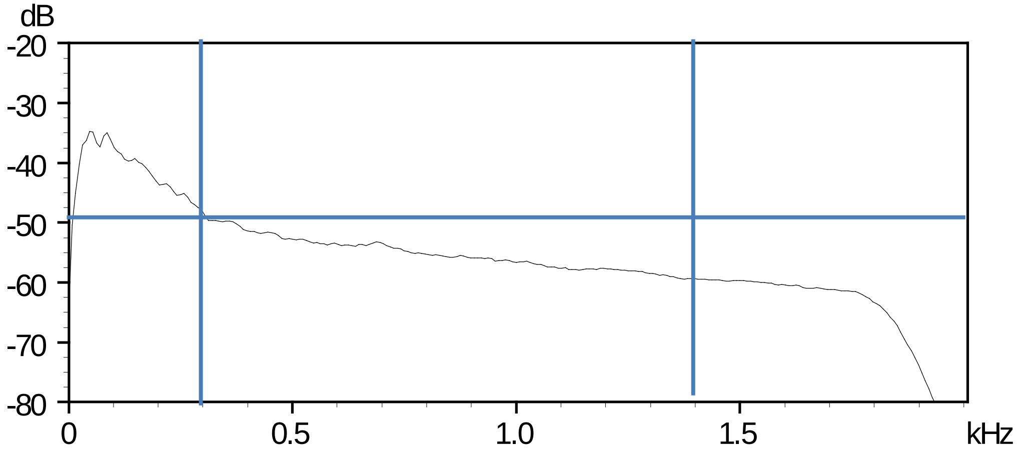

(f) low traffic roadside (LR)

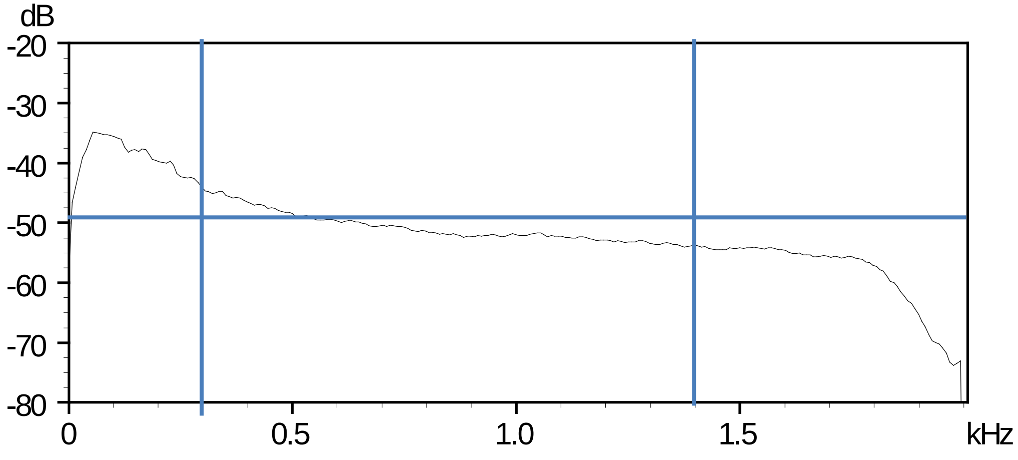

Audio 1. A representative test coo sound of *Chalcophaps indica*.

Audio 2. A representative test coo sound of *Geopelia striata*.

Audio 3. A representative test coo sound of *Spilopelia chinensis*.

Audio 4. A representative test coo sound of *Streptopelia orientalis*.

Audio 5. A representative test coo sound of *Streptopelia tranquebarica*.
